# Intimate partner violence and subsequent premature termination of exclusive breastfeeding: A cohort study

**DOI:** 10.1371/journal.pone.0217479

**Published:** 2019-06-10

**Authors:** Frederikke Kjerulff Madsen, Christina Elise Holm-Larsen, Chunsen Wu, Jane Rogathi, Rachel Manongi, Declare Mushi, Dan Wolf Meyrowitsch, Tine Gammeltoft, Geofrey Nimrody Sigalla, Vibeke Rasch

**Affiliations:** 1 Research Unit of Gynaecology and Obstetrics, University of Southern Denmark, Odense, Denmark; 2 OPEN, Odense Patient data Explorative Network, Odense University Hospital, Odense, Denmark; 3 Psychiatric Research Academy Odense, Odense, Denmark; 4 Institute of Public Health, Kilimanjaro Christian Medical University College, Moshi, Tanzania; 5 Department of Public Health, University of Copenhagen, Copenhagen, Denmark; 6 Department of Anthropology, University of Copenhagen, Copenhagen, Denmark; Wayne State University, UNITED STATES

## Abstract

**Objective:**

The objective of this study was to examine whether exposure to Intimate Partner Violence (IPV) is associated with premature termination of Exclusive Breastfeeding (EB). Per WHO recommendations, this was defined as ceasing breastfeeding or supplementing with other foods or liquids before the child was 6 months old.

**Method:**

It is a prospective cohort study set in Moshi, Tanzania consisting of 1128 pregnant women with live singleton births. Women were enrolled during pregnancy and followed up with interviews during pregnancy, after birth and 2–3 years postpartum, using structured questionnaires. Emotional, physical and sexual IPV exerted by the current partner was assessed at 34 weeks gestational age with WHO questionnaires. Months of EB was assessed 2–3 years postpartum. Premature termination of EB was defined as less than 6 months of EB. Analyses were made using a logistic regression model adjusted for maternal age, education, HIV-status, alcohol use during pregnancy and parity. Confounding variables were determined using a theoretical framework approach, i.e. a Directed Acyclic Graph model to minimize bias.

**Results:**

Women who were exposed to IPV had more than 50% higher odds of terminating EB before the child was 6 months old compared to women who were not exposed (aOR = 1.62, 95%CI: 1.27–2.06).

Women exposed to all three types of IPV had twice the odds of early termination of EB (aOR = 1.95, 1.12; 3.37). Furthermore, the odds were tripled if exposure happened specifically during the index pregnancy (aOR = 2.93 95%CI: 1.3; 6.6). Stratified analyses showed the most severely affected groups were the mothers older than 30 and those who gave birth to girls.

**Conclusions:**

The results indicated that exposure to IPV is associated with increased risk of premature termination of EB. The odds increase with multiple types of the IPV, especially when exposed during the index pregnancy.

## Introduction

Breastfeeding is a vital factor in maternal and child health. [1, 2] The World Health Organization (WHO) recommends initiating breastfeeding within 1 hour of birth, exclusive breastfeeding (EB) for 6 months and continued supplemental breastfeeding for 2 years.[3] Child benefits are reduced mortality, lower rates of infectious diseases and diabetes,[2, 4] and improved neurological and cognitive development.[5, 6] However, only 37% of children worldwide are exclusively breastfed for 6 months or more.[2]

Individual decision-making regarding breastfeeding is complex and influenced by biological, social and psychological factors.[7, 8] Research indicates that psycho-sociological factors have higher predictive roles for EB compared to socio-demographic factors. [9] A possible pathway is stress induced decrease in oxytocin impairing lactogenesis.[10–12] It was not until recently that intimate partner violence (IPV) was recognized as a potential risk factor leading to termination of EB.[13–15]

IPV is defined by the WHO as a “behaviour by an intimate partner or ex-partner that causes physical, sexual or psychological harm, including physical aggression, sexual coercion, psychological abuse and controlling behaviours”.[16] IPV is a global problem affecting one-third of women worldwide.[3] Few studies have investigated the effect of IPV on breastfeeding. Most studies are from America [14, 15, 17–20] or Asia.[13, 21–23] A systematic review from 2017[24] showed that just one African study has been published.[25]

Most studies were based on cross-sectional[13–15, 18, 19, 21–23, 25, 26] or case-control designs.[17] Only one study was prospective.[20]

Results from eight of twelve studies indicate that IPV has adverse effects on breastfeeding practices.[13, 15, 18–21, 23, 25] A cross-sectional multi-country study, had mixed findings depending on country.[25] Aside from adverse effects, they found that physical IPV in Tanzania and sexual IPV in Zambia surprisingly were associated with early initiation of breastfeeding and longer duration of EB, respectively. Four other studies found no significant association between IPV and breastfeeding.[14, 17, 22, 26]

None of eight studies addressing EB followed the population for long enough to determine if the children were exclusively breastfed for at least 6 months as is the recommendation by WHO. [13–15, 19–22, 25]

The aim of this study was to determine the effect of IPV on the duration of exclusive breastfeeding in Tanzanian women.

## Materials and methods

### Design

This was a prospective cohort study design. Data were collected from a population-based sample, which was part of a larger research project: “The Impact of Violence on Reproductive Health in Tanzania and Vietnam”.

### Sample (participants and settings)

All pregnant women, who attended antenatal care in Pasua and Majengo Health Clinics in Moshi, Kilimanjaro Region of Tanzania, were asked to participate.

Inclusion criteria were gestational age (GA) <30 weeks, determined by ultrasound.

Exclusion criteria were multiple pregnancies, plans to deliver outside of Moshi Municipality, pregnancies not resulting in live births and children or mothers who died during the study period.

### Measures

Data for this study was collected via five structured interviews. The first interview was at inclusion. The second interview was at 34 weeks GA. The third took place immediately after birth, the fourth interview was at 40 days postpartum and the final at 2–3 years postpartum. All data were double-entered.

Sociodemographic and reproductive data (age, parity, marital status and education level) were collected at the first interview. At the second interview, the women were asked about exposure to IPV, smoking and alcohol use and tested for HIV. The sex of the child and whether it was born preterm (GA <37 weeks) was registered at the third interview. At the fourth interview we asked for signs of postpartum depression using Edinburgh Postnatal Depression Scale (EPDS) with a score of >13 as a cut-off for probable depression. The tool has been validated in Africa, but not in Tanzania.[27] We collected data on the duration of breastfeeding at the final interview.

Recruitment was carried out between March 1^st^and December 31^st^, 2014. All interviews were done in private settings by experienced research assistants. Questionnaires and translations were pilot tested. Pilots were not included in the cohort.

#### Intimate partner violence

**Fig 1:**
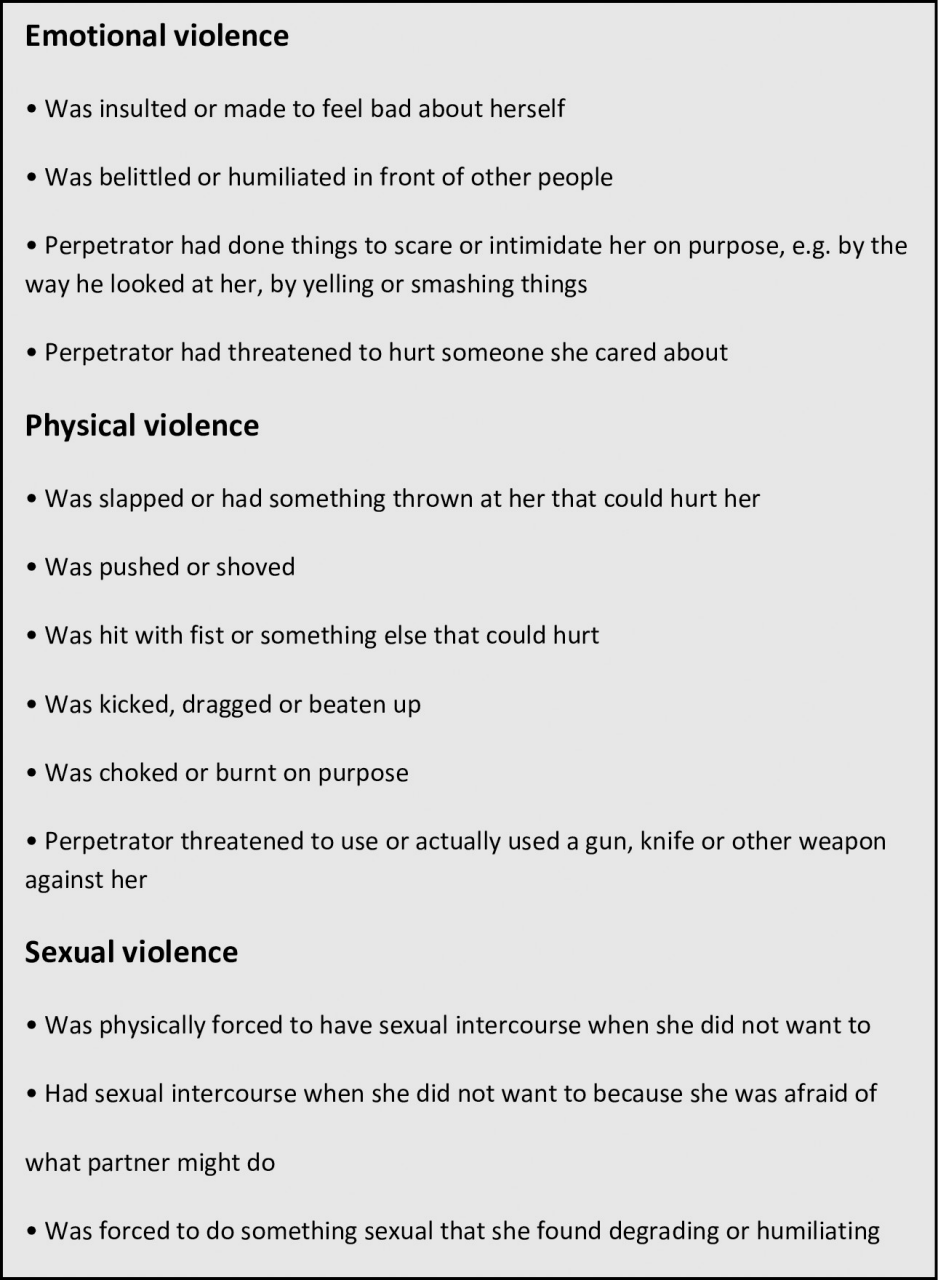
Definition of the different types of intimate partner violence.

The exposure to IPV was assessed at the second interview using a Kiswahili version of the questionnaire used in the WHO Multi-Country Study on Women and Domestic Violence against Women.[28] The questionnaire has three dimensions, pertaining to different types of violence; emotional, physical and sexual (Fig 1). Participants were asked about exposure to IPV both at any given time point in the relationship and specifically during pregnancy. Women who were exposed to ongoing physical or sexual IPV were referred to a separate counselor connected to the project, who had the possibility of referring to specific support services if the participant gave consent.

IPV was categorized in 5 different groups, who where not mutually exclusive: “At least one type of IPV”, “Emotional IPV”, “Physical IPV”, “Sexual IPV” and “All three types of IPV”.

The reference group was comprised of the women who had no exposure to IPV during the current relationship. All analyses were made twice–Once for women exposed at any point in the relationship and once for women exposed specifically during pregnancy.

#### Exclusive breastfeeding

The outcome of interest was premature termination of EB, defined as termination before the child was 6 months old. Total months of EB since birth was assessed 2–3 years postpartum and dichotomised premature termination if EB was less than 6 months.

### Statistical analyses

The distribution of cohort characteristics between exposed and un-exposed groups were compared using Pearson’s chi-square test. Fisher’s Exact test was used in the case of smoking, since less than 5 women smoked. Participants with missing values were excluded from the analysis.

Logistic regression was performed to estimate odds ratio (OR) with 95% confidence interval between IPV and premature termination of EB. We first estimated the crude OR and then adjusted for potential confounders, which were selected based on a priori assessment suggested by a directed acyclic graph (DAG),[29] including maternal age, education, HIV-status, alcohol use during pregnancy and parity(S1 Fig). As shown in the supporting information, we assume postpartum depression to be part of the pathway to reduced breastfeeding, and as such it is not adjusted for in the regression model. Instead it is addressed in the stratified analysis.

In order to determine if any groups were more vulnerable to the effects of IPV than others, we conducted a stratified analysis comparing women who were exposed to at least one kind of IPV at any point in their relationship to those who were not. We chose the variables maternal age, education, EPDS Score, HIV status and alcohol consumption as well as child sex for the stratification. The selection was made a priori based on clinical experience.

Statistical analyses were performed using STATA software package (version 15).

### Ethics

Ethical approval was granted by Research Ethics Review Committee of Kilimanjaro Christian Medical University College of the Tumaini University, Makumira, Tanzania.

Written permission was granted by Moshi Municipality Executive Director for carrying out research at Majengo and Pasua Health Clinics.

Oral consent was collected from all participants and documented by the research assistants carrying out the interviews.

## Results

**Fig 2:**
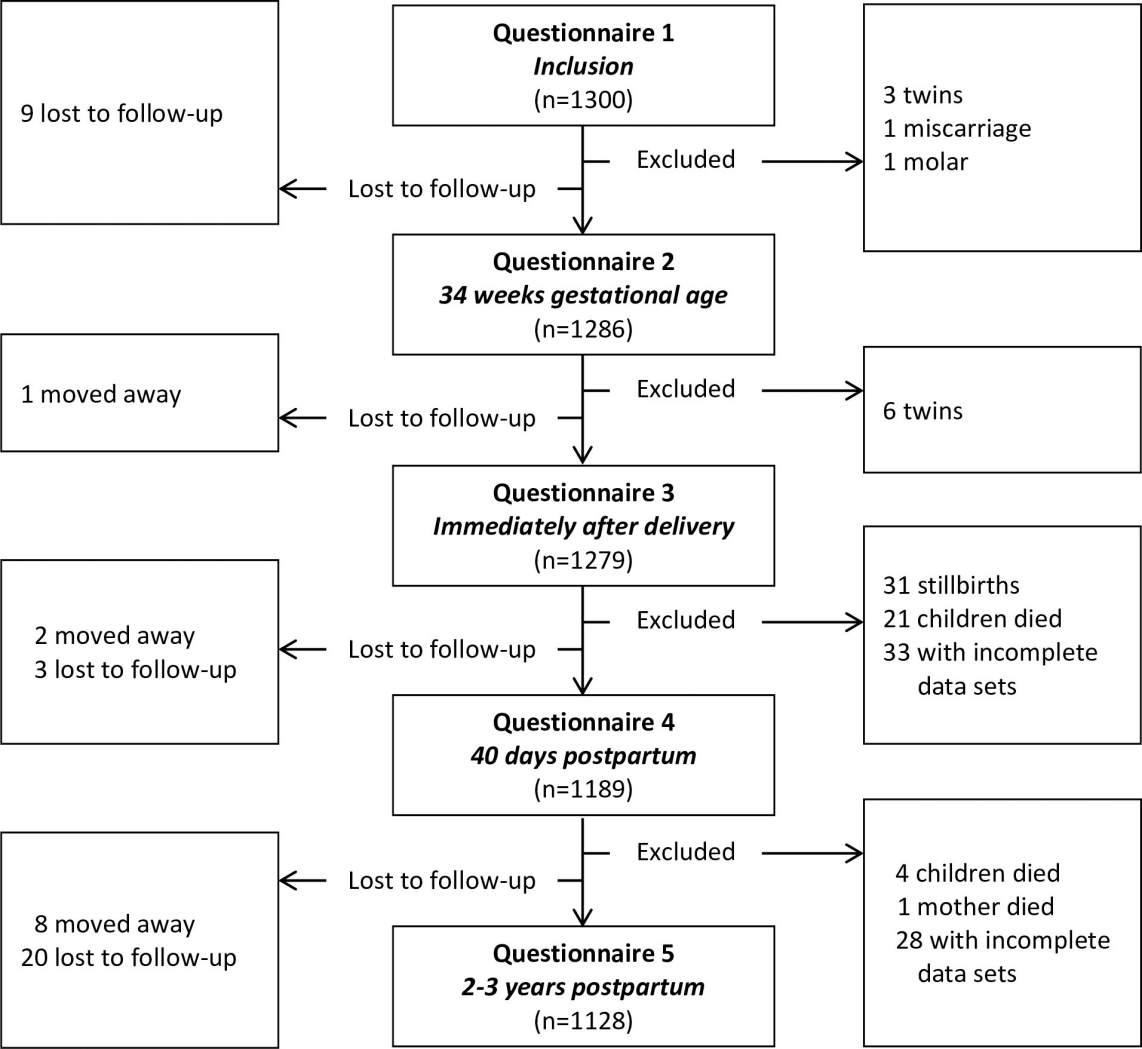
Inclusion flowchart.

A total of 1300 women fulfilled the inclusion criteria and 1128 (96.3%) mothers and their babies were included in the study (Fig 2).

Characteristics of the study participants and their children are summarized in Table 1. Most of the women were 20–30 years old, pregnant with their first or second child and had finished at least primary education. Women who were exposed to IPV were more likely to drink alcohol during their pregnancy, be HIV positive and show signs of postpartum depression.

**Table 1:** Descriptive characteristics of study participants.

Characteristics	No. of women included in analysis (% of those with characteristics)						P-value
	Total (n = 1128)		Reporting exposure to at least one kind of IPV** (n = 576)		Reporting no exposure to IPV (n = 552)		
**Maternal**							
Age ‡							
<20 years	137	(12.1%)	62	(10.8%)	75	(13.6%)	0.289
20–30 years	740	(65.6%)	388	(67.4%)	352	(63.8%)	
>30 years	251	(22.3%)	126	(21.9%)	125	(22.6%)	
Parity ‡							
0	459	(40.7%)	233	(40.5%)	226	(40.9%)	0.715
1	335	(29.7%)	183	(31.8%)	152	(27.5%)	
2	213	(18.9%)	100	(17.4%)	113	(20.5%)	
3	86	(7.6%)	42	(7.3%)	44	(8.0%)	
≥4	27	(2.4%)	14	(2.4%)	13	(2.4%)	
Education ‡							
Below primary education	24	(2.1%)	17	(3.0%)	7	(1.3%)	0.278
Primary education	725	(64.3%)	367	(63.7%)	358	(64.9%)	
Secondary education	323	(28.6%)	164	(28.5%)	159	(28.8%)	
Above Secondary education	56	(5.0%)	28	(4.9%)	28	(5.1%)	
Marital status (n) ‡							
Married	1011	(89.6%)	506	(87.8%)	505	(91.5%)	0.054
In a relationship, but not married	116	(10.3%)	69	(12.0%)	47	(8.5%)	
Smoking (n) †							
Any smoking during pregnancy	4	(0.4%)	0	(0.0%)	4	(0.7%)	0.057
Alcohol (n) ‡							
Any alcohol during pregnancy	128	(11.3%)	86	(14.9%)	42	(7.6%)	< .001*
HIV Status (n) ‡							
Positive	46	(4.1%)	35	(6.1%)	11	(2.0%)	0.001*
Signs of postpartum depression (n) ‡							
EPDS ≥ 13	138	(12.2%)	102	(17.7%)	36	(6.5%)	< .001*
**Child**							
Preterm birth							
<37 weeks ‡	65	(5.8%)	25	(4.3%)	40	(7.2%)	0.036*
Sex ‡							
Female	549	(48.7%)	288	(50.0%)	261	(47.3%)	0.361
Male	579	(51.3%)	288	(50.0%)	291	(52.7%)	

*P-value < 0,05

**Perpetrated by the current partner at any timepoint in the relationship

† P-value calculated with Fisher's Exact test

‡ P-value calculated with Chi2 test

The association between IPV and preterm birth in our cohort is described by Sigalla et al.[30]

**Fig 3:**
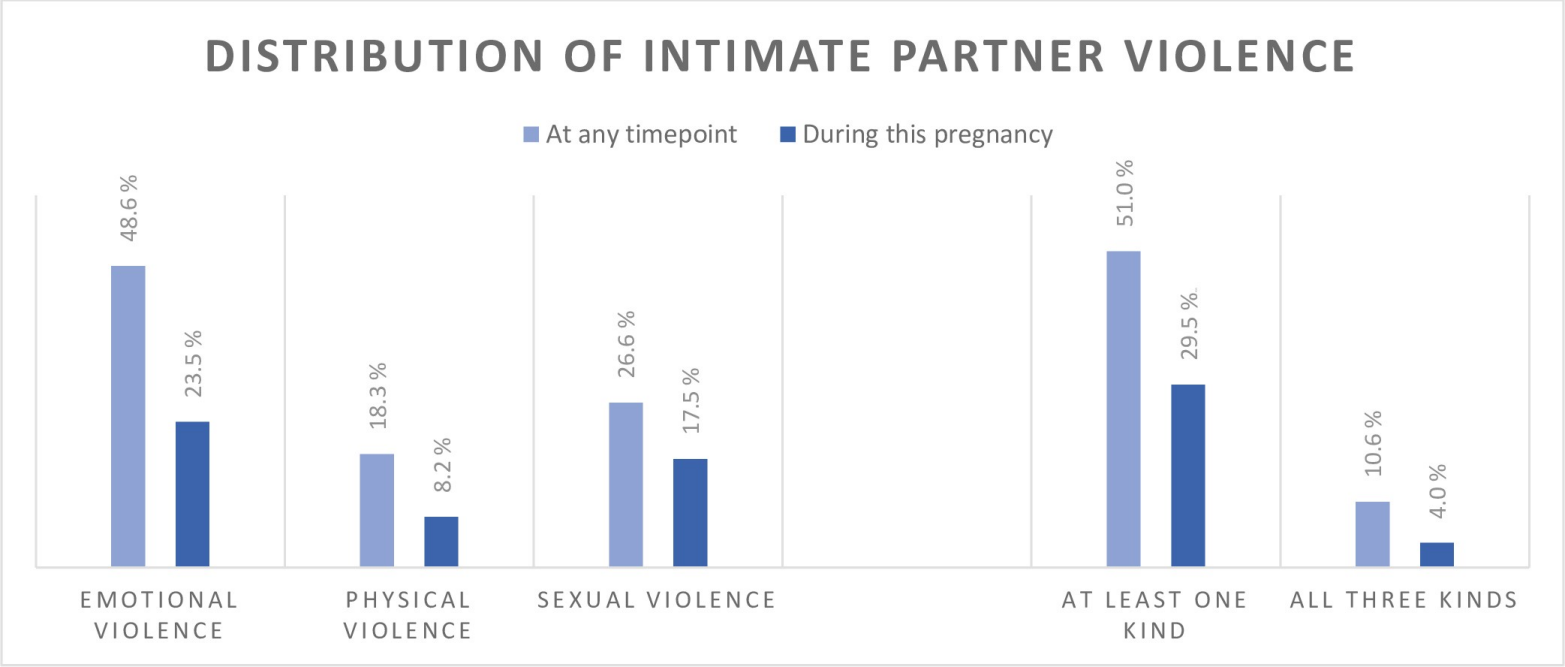
Distribution of intimate partner violence among women in the cohort. Total n = 1128.

Half the women were exposed to at least one kind of IPV during their current relationship. Almost a third were exposed specifically during pregnancy. The distribution of subtypes of IPV is shown in Fig 3

Table 2 displays the odds of ceasing EB before 6 months as a function of IPV exposure. Women exposed to IPV at any point in their relationship had more than 50% higher odds of premature termination EB compared to the women who were not exposed. This was the case for all three types of violence. Women who were exposed to all three types had almost double the odds of termination exclusive breastfeeding before 6 months.

**Table 2:** Associations between exposure to intimate partner violence (IPV) and termination of exclusive breastfeeding before 6 months.

Reported exposure during current relationship	Premature termination of exclusive breastfeeding (n = 577)						
	No.	Unadjusted OR(95%CI)			*Adjusted OR(95%CI)		
At least one type of IPV	326	**1.56**	(1.24;	1.98)	**1.61**	(1.26 ;	2.05)
Emotional IPV	297	**1.57**	(1.23 ;	2.00)	**1.61**	(1.26 ;	2.07)
Physical IPV	67	1.41	(0.95 ;	2.08)	**1.53**	(1.01 ;	2.31)
Sexual IPV	111	**1.50**	(1.08 ;	2.07)	**1.50**	(1.07 ;	2.09)
All three types of IPV	39	**1.80**	(1.07 ;	3.04)	**1.93**	(1.11 ;	3.34)
Reported exposure during pregnancy	No.	Unadjusted (95%CI)			*Adjusted OR(95%CI)		
At least one type of IPV	182	1.22	(0.94 ;	1.58)	1.25	(0.96 ;	1.62)
Emotional IPV	131	1.16	(0.87 ;	1.55)	1.23	(0.91 ;	1.65)
Physical IPV	42	1.47	(0.90 ;	2.40)	1.68	(1.00 ;	2.82)
Sexual IPV	97	1.38	(0.99 ;	1.94)	1.35	(0.96 ;	1.91)
All three types of IPV	23	**2.59**	(1.18 ;	5.66)	**2.87**	(1.27 ;	6.46)

Total sample size n = 1128. The reference category is women, who were not exposed to any kind of IPV (n = 552).

*Adjusted for maternal age, education, HIV status, alcohol use during pregnancy and parity

For the women who were exposed specifically during the index pregnancy, the adverse effect remained significant only in cases of triple exposure, where the women had three times increased odds of premature termination of EB.

The data for the stratified analysis is presented in Table 3. In cases with exposure to all three types of IPV, some of the analysed categories did not contain enough women-offspring pairs to meet the assumptions for logistic regression. We chose to display the data in the table, but they should be tentatively reviewed.

**Table 3:** Stratified analyses of associations between exposure to intimate partner violence (IPV) and termination of exclusive breastfeeding before 6 months.

	Premature termination of exclusive breastfeeding (n = 577)							
Indicator	Exposed to at least one type of IPV*				Exposed to a combination of all three types of IPV*			
	No.	**Adjusted OR(95%CI)			No.	**Adjusted OR(95%CI)		
Overall	326	**1.62**	(1.27 ;	2.06)	39	**1.95**	(1.12 ;	3.37)
**Child sex**								
Female	177	**2.05**	(1.44 ;	2.91)	19	**2.33**	(1.02 ;	5.32)
Male	149	1.27	(0.91 ;	1.77)	149	1.63	(0.77 ;	3.42)
**Maternal signs of postpartum depression**								
EPDS Score < 13	266	**1.58**	(1.22 ;	2.05)	25	1.52	(0.80 ;	2.87)
EPDS Score ≥ 13	60	1.87	(0.84 ;	4.16)	14	3.49	(0.95 ;	12.88)
**Maternal HIV status**								
HIV Negative	316	**1.62**	(1.27 ;	2.07)	36	**1.95**	(1.10 ;	3.46)
HIV Positive	10	1.15	(0.24 ;	5.59)	3	1.84	(0.19 ;	17.79)
**Maternal alcohol intake during pregnancy**								
No alcohol intake	275	**1.65**	(1.28 ;	2.13)	32	**2.26**	(1.23 ;	4.16)
Any alcohol intake	51	1.27	(0.58 ;	2.79)	7	0.98	(0.25 ;	3.85)
**Maternal age**								
<20 years	29	1.16	(0.57 ;	2.34)	4	N/A		
20–30 years	216	**1.51**	(1.12 ;	2.03)	20	1.21	(0.62 ;	2.38)
>30 years	81	**2.31**	(1.34 ;	3.97)	15	**4.57**	(1.35 ;	15.40)
**Maternal education**								
Primary or lower	201	**1.54**	(1.14 ;	2.07)	29	1.61	(0.87 ;	2.99)
Secondary or higher	113	**1.75**	(1.15 ;	2.66)	10	**4.19**	(1.09 ;	16.12)

Total sample size n = 1128. The reference category is women, who were not exposed to any kind of IPV (n = 552)

*Perpetrated by the current partner at any time point in the relationship

**Adjusted for maternal age, education, HIV status, alcohol use during pregnancy and parity

The most severely affected groups were the mothers older than 30 and those who gave birth to girls. Mothers of girls had more than twice the odds of premature termination of EB if they were exposed to at least one type of IPV. Women over thirty had twice the odds of premature termination of EB and more than 4 times the odds if they were exposed to all three types.

Postpartum depression in Table 3 was demonstrated to be a modifying factor in Table 3. However, it was not significantly associated with premature termination of EB. Analyses are published elsewhere.[31]

## Discussion

### Main findings

To the best of our knowledge, this is the first longitudinal study to explore the association between IPV and EB in Africa and the first study to provide the 6 months’ time perspective. It provides strong evidence on the suspected adverse effects of IPV suggested by other studies.[13, 15, 18–21, 23, 25] Women who reported exposure to at least one type of IPV at any point in the relationship had more than 50% higher odds of terminating EB before the child was 6 months old. When exposed to all three types of IPV, women had twice the odds of premature termination of EB when exposed at any point in their relationship. When exposed to all three types of IPV during the index pregnancy the odds were tripled. As the odds increase with the severity of IPV, a dose-response relationship could be present. However, the current tools available to assess IPV do not allow for an appropriate analysis to ascertain that.

The results of the stratified analysis demonstrated that associations between IPV and EB are complex with several modifying factors. Knowing which subgroups of mothers are particularly vulnerable, enables health workers to target interventions in settings with limited resources.

While maternal education, alcohol use during pregnancy and signs of postpartum depression all influenced the outcome, the most prominent modifications were seen with mothers who are more than 30 years old and who gave birth to girls. This interesting finding may stem from several reasons. Boys might be better at breastfeeding, mothers might perceive them to be either more capable or more in need of exclusive breastfeeding or there might be societal factors deeming girls less desired by a violent partner, hence giving birth to a girl might escalate violence in the family, increasing the adverse effect.[32] That the odds of early weaning increase with maternal age, suggests more resilience in younger mothers. Health professionals should be aware of older mothers and mothers of girls and watch out for signs of abuse.

### Strengths and limitations

The main strengths of this study were the longitudinal design and high follow-up rate. With more than 90% of participants completing all interviews, the risk of attrition bias is negligible, and we believe selection bias has been minimized, since all women who met the wide inclusion criteria, agreed to participate.

However, there are some limitations to the study. Firstly, inquiring about IPV might inspire the exposed mothers to react to the violence and adopt protective measures, thereby limiting the exposure causing an underestimation of the adverse effect. While IPV is a complex exposure to measure, our findings on IPV prevalence are in line with what we expected from previous studies.[33, 34]

Secondly, the WHO tool we used to assess IPV does not allow for a reliable assessment of continued IPV. Therefore, we do not know if the violence was ongoing for the participants at follow-up.

Thirdly, there is no failsafe way to measure EB. The two most common approaches are either the 24-hour recall method or the “since birth” method.[35, 36] Both come with the risk of over- and underreporting. We believe the “since birth” method provides the most reliable results. However, the time elapsed before the follow-up interview introduces an increased risk of information bias. The method of distant recall is not optimal. For practical and financial reasons, a close follow-up was not possible in this study.

There is a need to develop a validated tool to assess breastfeeding practices to increase the quality of future studies on the subject.

### Interpretation

Our findings are consistent with the results from most studies on IPV and breastfeeding despite the different outcome measures used. Other studies examine either initiation, intention to breastfeed or EB in the past 24 hours or since birth, but not after the age of 6 months.[13, 15, 18–21, 23, 25] Our findings are in keeping with the patterns observed in previous research on breastfeeding outcomes and IPV. It also provides a significant addition to the current literature by virtue of the longitudinal 6 months perspective.

Our findings provide evidence for the deficit hypothesis,[37] which proposes that women exposed to IPV are less inclined to or less capable of breastfeeding their children. There is qualitative evidence that sexually abused women could attach negative sexual associations to their breasts and concurrently be less willing to breastfeed.[38] Women suffering from IPV are also likely to engage in negative coping behavior[39] and might be physically, psychologically and cognitively impaired.[40] They are also more likely to suffer from postpartum depression compared to their unexposed peers.[41–43]

The mechanism of breastfeeding contains both physiological and emotional elements which can be disrupted under stress. Animal studies have demonstrated how stress lowers lactogenesis by decreasing oxytocin levels.[11] Literature on the human stress response and lactation is sparse; however experimental studies suggest the same mechanisms might apply.[10, 12]

Although, the observed association between IPV and breastfeeding might reflect a causal link between the two factors, it is also possible that both IPV and premature termination of breastfeeding are independent proxy indicators for a dysfunctional family pattern. Besides the physiological stress response, we know self-efficacy and social support are paramount to breastfeeding and are likely to be impaired by IPV.[44] As described in another paper, mothers exposed to IPV in our cohort had significantly less social support than mothers without exposure.[45]

The only study with conflicting findings was Misch et al, who found IPV to have a positive effect on breastfeeding in Tanzanian and Zambian women, as mentioned in the introduction.[25] They proposed the effect could be due to compensatory behavior, where the mother used breastfeeding as a coping mechanism to deal with violence. This compensatory hypothesis was proposed by Levendosky et al. in 2003 in a study on the maternal relationship with pre-school children in families with domestic violence. The study did not address breastfeeding specifically.[46] The adverse effects of IPV stress the need for interventions to aid the mothers exposed to IPV. Violence is a convoluted cultural phenomenon making the exposure difficult to tackle. While a diversity of adverse effects of IPV by now are well documented, an effective intervention has yet to be developed, possibly because most attempts are aimed at victims instead of perpetrators.[47] Despite the lack of effective interventions several studies recommend screening for IPV during pregnancy.[16, 47] The salient reason for this is that awareness is the first step to leave a violent relationship. Furthermore, pregnant women are in a situation where they are uniquely motivated to seek help.[47]

Further research to find effective interventions against IPV, preferable aimed at the perpetrators, is vital. Right now, however, there is an acute need for assistance for women trapped in violent relationships. Safe exit possibilities are needed to ensure their continued ability to provide optimal care for their children.

## Conclusion

The present study provided strong evidence to support that exposure to IPV increases the odds of terminating EB before the recommended 6 months. This calls for initiatives and policies to prevent IPV and help the women affected.

An effort aimed at detecting pregnant victims of IPV and providing them with comprehensive ante- and postnatal care has the potential of optimizing breastfeeding for women and their children, thereby reducing child morbidity and mortality significantly.

## Supporting information

S1 Fig Theoretical framework. Directed acyclic graph.
(TIF)

S2 Fig Source data. (DTA)
