# Supplementary figures and images for: Intimate partner violence and subsequent premature termination of exclusive breastfeeding: A cohort study
Source: PLoS One. 2019 Jun 10;14(6):e0217479. doi: 10.1371/journal.pone.0217479 (PMC6557484; doi:10.1371/journal.pone.0217479)

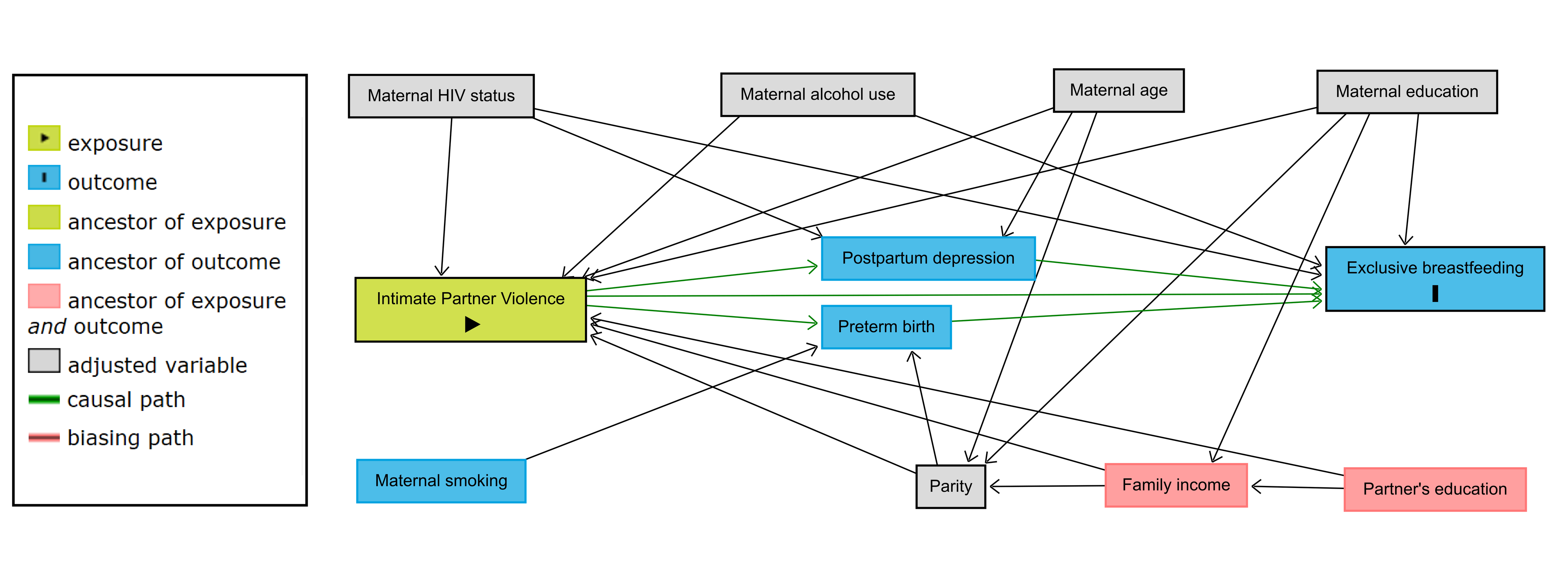

Supplement: S1 Fig — Directed acyclic graph. (TIF) [file pone.0217479.s001.tif]
